# The role of artificial intelligence in paediatric neuroradiology

**DOI:** 10.1007/s00247-022-05322-w

**Published:** 2022-03-26

**Authors:** Catherine Pringle, John-Paul Kilday, Ian Kamaly-Asl, Stavros Michael Stivaros

**Affiliations:** 1 Children’s Brain Tumour Research Network (CBTRN), Royal Manchester Children’s Hospital, Manchester, UK; 2 Division of Informatics, Imaging, and Data Sciences, School of Health Sciences, Faculty of Biology, Medicine, and Health, University of Manchester, Manchester, UK; 3 The Centre for Paediatric, Teenage and Young Adult Cancer, Institute of Cancer Sciences, University of Manchester, Manchester, UK; 4 Department of Paediatric Radiology, Royal Manchester Children’s Hospital, Central Manchester University Hospitals NHS Foundation Trust, Oxford Road, Manchester, M13 9WL UK; 5 The Geoffrey Jefferson Brain Research Centre, Manchester Academic Health Science Centre, University of Manchester, Manchester, UK

**Keywords:** Artificial intelligence, Children, Machine learning, Magnetic resonance imaging, Neuroradiology, Radiogenomics

## Abstract

Imaging plays a fundamental role in the managing childhood neurologic, neurosurgical and neuro-oncological disease. Employing multi-parametric MRI techniques, such as spectroscopy and diffusion- and perfusion-weighted imaging, to the radiophenotyping of neuroradiologic conditions is becoming increasingly prevalent, particularly with radiogenomic analyses correlating imaging characteristics with molecular biomarkers of disease. However, integration into routine clinical practice remains elusive. With modern multi-parametric MRI now providing additional data beyond anatomy, informing on histology, biology and physiology, such metric-rich information can present as information overload to the treating radiologist and, as such, information relevant to an individual case can become lost. Artificial intelligence techniques are capable of modelling the vast radiologic, biological and clinical datasets that accompany childhood neurologic disease, such that this information can become incorporated in upfront prognostic modelling systems, with artificial intelligence techniques providing a plausible approach to this solution. This review examines machine learning approaches than can be used to underpin such artificial intelligence applications, with exemplars for each machine learning approach from the world literature. Then, within the specific use case of paediatric neuro-oncology, we examine the potential future contribution for such artificial intelligence machine learning techniques to offer solutions for patient care in the form of decision support systems, potentially enabling personalised medicine within this domain of paediatric radiologic practice.

## Introduction

In recent years interest has focussed on the use of radiomics, the extraction of vast amounts of quantitative features from standard medical imaging (via data-characterising algorithms) to improve the radiophenotyping of neurologic disease. The process of enabling computers to perform this task and indeed learn from such data to facilitate clinical decision-making is often referred to as artificial intelligence (AI). Such approaches have been employed in multiple applications in paediatric neuroradiology, particularly in regard to neuro-oncology [1]. This article reviews the techniques used to underpin AI in paediatric neuroimaging. We examine the strengths and weaknesses of such techniques and highlight specific applications within the gamut of paediatric neuroimaging, in which they have been employed. In regard to brain tumours in children, we discuss why such systems are required going forwards and, using exemplars within paediatric neuro-oncological imaging, we examine what the future of such systems might hold.

## Machine learning techniques

Artificial intelligence is the concept of making a computer system “clever” and, in a radiologic setting, able to make decisions based on the imaging data presented to it. Machine learning refers to how we afford computers the opportunity to do this. Rather than having to program a system to perform a decision-making task in paediatric neuroradiology, machine learning is where effective computer algorithms are developed that learn from experience (like a human) and create models, with associated decision-making skills, from a given body of supplied data. In the imaging domain, such data incorporate the imaging study itself.

There has been rapid proliferation in the application of machine learning techniques in health care over recent years, secondary to the evolution of electronic health care records, increasing patient data accrual, and a growing interest in health informatics [2, 3]. Machine learning techniques are often applied to conditions where traditional statistical analysis might not generate accurate results. An example of this would be modelling large feature-rich datasets from imaging studies with relatively small sample sizes of patients, where there is a non-Gaussian distribution and class imbalances (e.g., relative numbers of specific tumour type) [4, 5]. Several machine learning applications in health care have achieved physician-level accuracy when tasked with diagnosing skin, breast and chest lesions using imaging data alone [6–9].

Machine learning models tend to follow two general schema, supervised or unsupervised approaches, with the former defined by whether they “learn” from a provided and characterised training dataset [10]. Unsupervised learning techniques do not use training data but rather draw inferences from unlabelled data without any pre-defined outputs. Supervised learning algorithms, when applied to relatively small data sets as seen in paediatric neuroradiologic settings, can be prone to overfitting [11]. They are, however, on the whole, advantageous in that the categories and classifiers generated are interpretable by humans, based on the labelled data used to train them. We continue by examining some supervised machine learning techniques and looking at specific paediatric neuroimaging applications underpinned by them.

## Supervised machine learning techniques

### Artificial neural networks

#### Technique

Artificial neural networks (ANNs) are so termed because of their resemblance to a biological neural network. They are composed of a network of nodes that are interlinked by connections, which are weighted. This connection weighting increases or decreases the strength of the connection between nodes and auto-adjusts as the model learns from its training data. Typically, ANNs consist of layers of node networks, with the output generated after data have traversed the network and reached the last layer [12, 13].

#### Applications

**Fig. 1:**
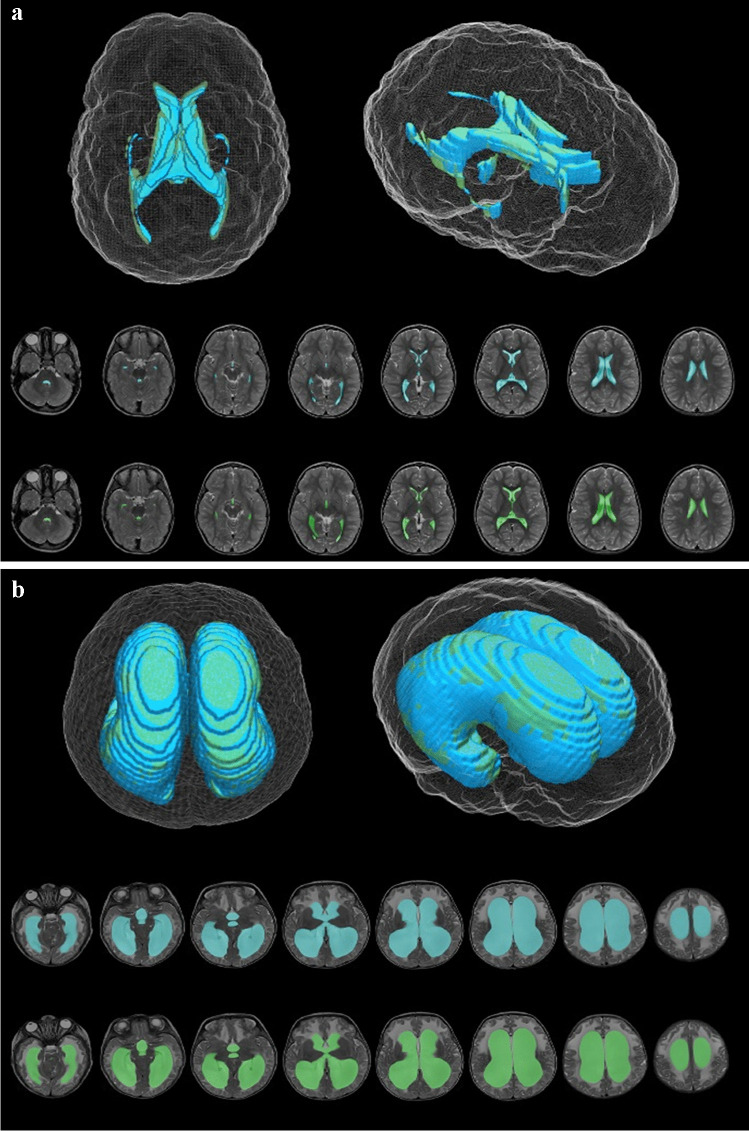
Artificial neural networks. **a, b** Deep learning model (*blue*) and ground truth manual (*green*) segmentation of a representative control (**a**) and hydrocephalus (**b**) using axial T2-weighted MR images. Reproduced from [19]

Artificial neural networks have received significant exposure of late given their success with, for example, chest radiograph characterisation, often being described as “big-data” analysis. However, only moderate successes have been observed in brain tumour imaging, predominantly in the adult neuro-oncology sphere [14–18]. A major issue with their application in paediatric neuroimaging is that the radiologic analysis of “big-data” is far removed from the requirements of machine learning within the paediatric neuroimaging domain. For example, a chest radiograph is a single image for which hundreds of thousands of training images are available, as opposed to paediatric brain imaging, for which the training dataset is several orders of magnitude smaller in number of images and is far more heterogeneous. In some paediatric neuroradiologic applications, ANNs have had good success in a targeted form. This is best illustrated in the determination of ventricular size to stratify children into normal or hydrocephalic. Quon et al. [19] were able to undertake such an analysis with an accuracy score of 94.6% in hydrocephalus vs. 85.6% in controls based on a training set of T2-weighted MR images from approximately 399 children (Fig. 1). Other groups have had similar success in paediatric hydrocephalus with evolutionary changes in ANN techniques [20]. ANN performance can be enhanced through the application of attention-based neural networks, which are capable of producing spatial maps that highlight regions of interest for either object detection or interpretation. This approach is useful when dealing with large volumes of complex imaging data, such as MRI brain data sets. Attention-based ANNs applied to fetal MRI data sets were successful in aging fetal brain and detecting anomalies, achieving area-under-the-curve (AUC) values of up to 90% [21, 22]. Similarly, convolutional neural networks (CNNs) have also successfully segmented fetal MRI, improving the antenatal diagnosis of spina bifida [23].

### Support vector machines

#### Technique

Support vector machines (SVMs) analyse and group labelled input data into classes, separated by the widest plane (support vector). They are often used in cases where there is a non-linear relationship between data, and, as such, a separation plane is not easily distinguishable. They tend to be used to categorise data into binary groups, e.g., “is this tumour a pilocytic astrocytoma or a medulloblastoma?” However they can be nested to allow for more complex decision-making.

#### Applications

**Fig. 2:**
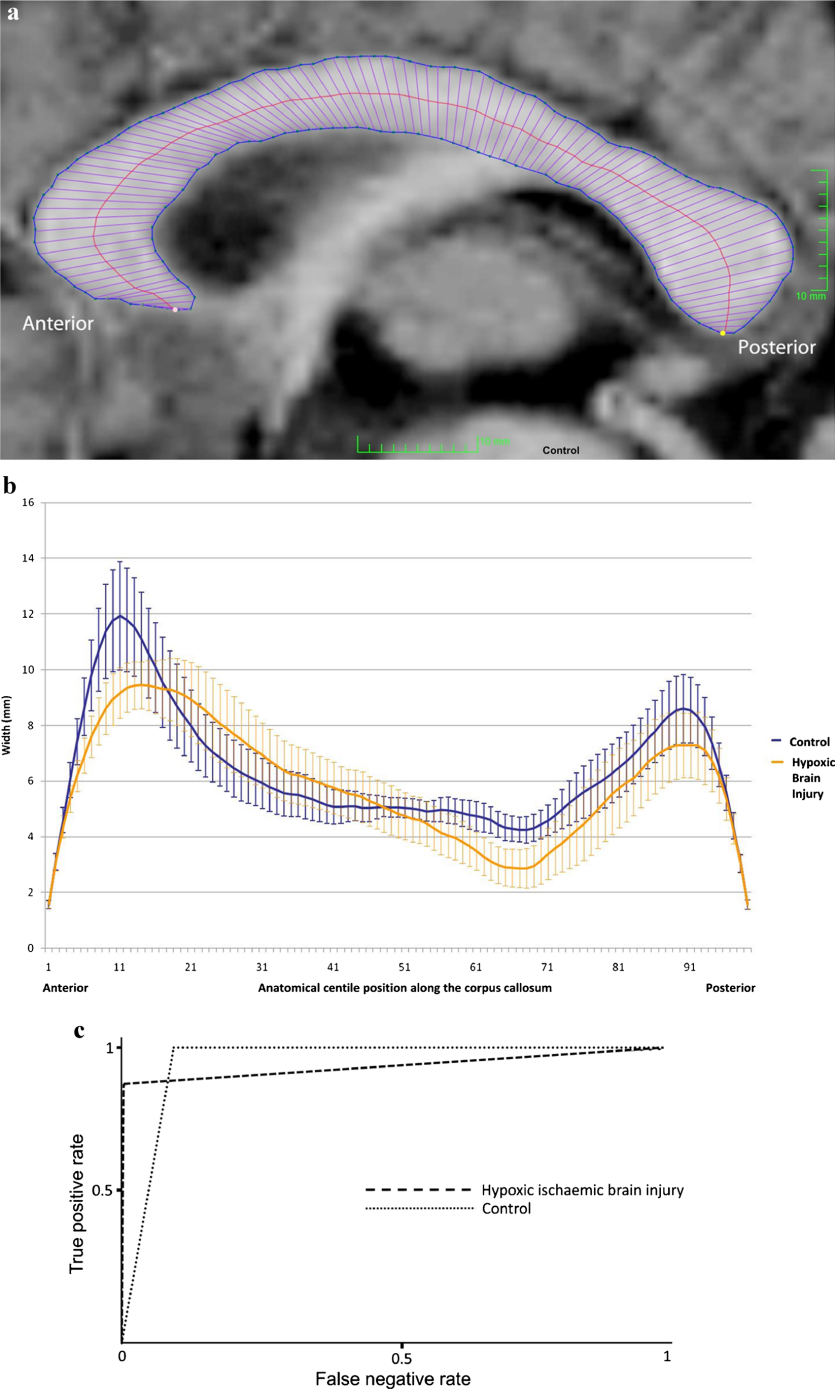
Support vector machine (SVM) stratification. **a** Midline sagittal T1-weighted MR image shows a normal corpus callosum in a 6-year-old age-matched male control. The image also shows the placement of regions of interest and 99th-percentile widths. **b** The width profiles (95% confidence interval of the mean) for each centile generated for the control cases (*blue*) and the age-matched cases of profound hypoxic–ischaemic brain injury (*yellow*). **c** SVM stratification performed on the imaging dataset for each participant with classification into one of two groups: (1) hypoxic–ischaemic brain injury or (2) developmental delay control. Receiver operator characteristics curves show classification (correct classification = true positive) into either the hypoxic–ischaemic brain injury group or the control group. Note the high degree of stratification, with an area under the curve of over 95% relating to both groups. This demonstrates the power of this technique when applied to this particular imaging metric. As such, it points towards machine learning callosal analysis in translational clinical and academic applications. Reproduced from [24]

A significant proportion of paediatric neuroimaging relates to neonatal imaging and attempts to generate outcome prediction relating to possible neuro-trauma. The authors of this paper had some success in this regard using the thickness of the corpus callosum (measured on a single sagittal T1-weighted MR image), to determine whether a child had suffered from hypoxic–ischaemic brain injury [24]. Principal component analysis identified that children who had suffered from such an injury were statistically more likely to have thinning of the posterior body of their corpus callosum. This was as hypothesised, given the radiation of white matter fibres through this region of the corpus callosum from the perirolandic cortex, which represents an area of the brain often seen to be damaged in children with spastic cerebral palsy as a result of acute profound hypoxic–ischaemic brain injury. SVM stratification of children into either normal or hypoxic–ischaemic brain injury based on corpus callosum widths was then possible with an accuracy of 95% (Fig. 2; [24]). Raji et al. [25] used a similar technique to analyse adolescents who had suffered from traumatic brain injury (TBI) on the basis of edge density imaging — again, using SVMs to stratify into normal or in this case mild TBI. This technique, which yielded an accuracy of 94% (with sensitivity of 79% and specificity of 100%), was able to outperform neurocognitive testing in this regard [25]. SVMs have also successfully classified a range of MRI abnormalities on fetal brain, including functional connectivity, brain maturity and severe fetal abnormalities, with accuracies of 79–84% [26–28].

### Decision trees

#### Technique

Decision trees process observations about an object (branches) into conclusions about an item’s target value (leaves). Decision tree analysis is a decision modelling process with weighting of the branches at each step of the tree. This is an open process, in contrast to the black box ANNs discussed earlier, in that the branches and weights are visible and interpretable to end users. By way of example, in brain tumour assessment, the branches could consist of the anatomical location of a suspected tumour and advanced metric MRI features, with the final leaf representing the predicted tumour diagnosis. Decision trees also offer feature selection, in that features can be removed to improve the efficiency of the model. Decision trees are able to process large volumes of both numerical and categorical data, which is imperative in modern health care. However, decision trees are susceptible to small alterations in the training data set causing drastic changes in the final classification, while overly complex trees can lead to overfitting of training data and the inability to process novel data correctly [29, 30].

#### Applications

Decision tree analysis has also been used in the field of neonatal imaging to aid in diagnosis and prognostication. Liu et al. [31] used such a technique to analyse features on MRI to better characterise between acute bilirubin encephalopathy and normal myelination patterns. This study was interesting for two reasons. First, it included both radiomic data as well as radiologist-defined imaging features. Hence it is an exemplar of harnessing the power of the radiologist in conjunction with computer-aided imaging analysis. Second, it compared machine learning techniques and found that in this setting the AUC of the decision tree analysis was 94.6%, compared to 93.1% for SVM, given the same dataset. The latter point illustrates that in most imaging AI applications, the application of multiple machine learning techniques is often necessary to determine the best approach to the data being modelled. A one-size-fits-all paradigm is not optimal going forwards as more of these systems are developed for clinical applications in paediatric neuroradiology.

Decision trees have also been used in another important role of machine learning in paediatric neuroimaging — that of data analysis to inform on neuroimaging in clinical trials. As more and more studies become reliant on multi-parametric data, we require more complex methodologies to analyse and model the resultant imaging findings. One example is the authors’ decision tree classifier, used in a randomised controlled trial in children with neurofibromatosis type 1 (NF1)-related autism who were being treated with simvastatin [32]. In this work the neuroimaging included multi-voxel gamma aminobutyric acid (GABA) spectroscopy, perfusion imaging, diffusion tensor imaging and resting-state functional MRI as well as standard anatomical assessment, an exemplar of the types of complex imaging studies that are becoming ever more commonplace as our imaging sequences develop. Making sense of these data presents a real challenge, which AI can help facilitate. In the simvastatin study [32], a random forest classifier was able to classify children into those who had been treated with simvastatin versus controls with an accuracy of 79%. This is potentially indicative of the gains such applications will afford us in the future.

### Naïve Bayesian classifiers

#### Technique

Naïve Bayesian classifiers are probabilistic classifiers that assume that all imaging variables available in a study are independent of one another, when generating the probability that a diagnosis is of a particular type. They have the advantage that they require relatively small training data sets, but in general terms they are currently thought to perform comparatively less well against other techniques.

#### Applications

The diagnosis of autistic spectrum disorder is made based on clinical neuropsychological/psychiatric assessments in children. These children often have brain scans to look for underlying structural or associated germline conditions that might present with autistic spectrum disorder, e.g., single aberrant gene conditions such as NF1 or tuberous sclerosis. The majority of children with autistic spectrum disorder, however, do not have such underlying conditions and thereby have idiopathic autistic spectrum disorder. To date, such cases were not thought to have an underlying structural aetiology that was identifiable on imaging. Chen et al. [33], however, took structural MRI data in such children and through the application of a naïve Bayesian classifier made optimal use of these data by analysing a three-dimensional histogram of oriented gradients (a form of image processing descriptor) in various regions of the brain, e.g., the frontal gyrus and hippocampus, amongst others; this preliminary work, which also analysed data from multiple centres, achieved a maximal AUC accuracy of 84.9% in regard to identifying MRI-based biomarkers to differentiate children with autistic spectrum disorder from normal controls. This is an exemplar of the use of AI to extract maximal information from an imaging dataset in a manner that had not been achievable.

Naïve Bayesian classifiers have also been successful when applied to MR imaging of another rare cohort with a small available imaging training data set: children with antenatally diagnosed fetal abnormalities. Naïve Bayesian classifiers were capable of classifying abnormalities including agenesis of the corpus callosum, Dandy-Walker variants, colpocephaly, mega-cisterna magna, cerebellar hypoplasia and polymicrogyria with accuracies of 63–91% [27].

### Linear discriminant analysis

#### Technique

Linear discriminant analysis (LDA) classifies cases by identifying relationships between combinations of features that can discriminate two or more types using continuous independent variables, which are in turn analysed against a final categorical dependent variable. However, LDAs assume normal distribution of data with homogeneous variance; this can be an unsafe assumption to make in small cohorts of a rare disease process such as those in children’s neuroimaging. However, there have been some successful applications in the neonatal setting.

#### Applications

Gui et al. [34] used an LDA to compare the longitudinal changes in regional brain volumes (including cerebrospinal fluid, cerebellar, unmyelinated white matter and grey matter volumes) in preterm infants (26–36 weeks of gestational age at birth), then again at term equivalence. Using this LDA approach, they then modelled these volumes and their changes, with the addition of various perinatal factors such as sepsis or a persistent ductus arteriosus. The models derived used outcome measures of both motor and neurocognitive function at 18 months and 5 years of age. This is important in regard to the incorporation of clinical factors into the imaging model. The ability of machine learning techniques to integrate such data is a major advance in our ability to prognosticate for children beyond what would be possible using imaging alone, especially given the fact that most of these childhood diseases represent complex biological systems that can only be described in terms of multiple different domains of data.

### Unsupervised learning algorithms

Any discussion regarding AI in paediatric neuroradiology must include a review of unsupervised learning algorithms, although, as mentioned, the relatively small number yet feature-rich datasets in this clinical domain result in limited success in this domain of radiologic practice. K nearest neighbours (kNNs) are pattern recognition systems that classify a new tumour into a previously recognised type within which the majority of its features would fall [35]. Whilst kNNs are an appealing technique because no assumptions are placed on the data and the system constantly evolves, they only work optimally when dealing with small numbers of input variables (unlike the imaging datasets discussed here). Whilst kNNs have been applied to hydrogen 1 (^1^H) MR spectroscopy data to classify adult brain tumours [36, 37], they have had little success in the analysis of paediatric brain tumours and no significant application in any other domains of paediatric neuroradiology.

## Potential pitfalls with machine learning techniques

As stated, machine learning limitations include overfitting of data and sensitivities to outliers. At an end-user level, clinicians might be reluctant to accept the output of a black box classifier system such as an advanced neural network because the decision-making process is not fully visible. This is perhaps reflected in the low translational uptake of decision support systems into routine clinical practice. There is also a lack of standardised legal regulation as exists for medical devices, and there are no clear legal guidelines about independent mathematical interrogation and validation of outputs generated by AI systems. These factors also hamper the acceptance of such systems into routine clinical practice [38]. Finally there is the ethical consideration regarding the use of large volumes of patient data and integrating said data from multiple patient sources into one repository and an analysis system built upon such a data construct. Problems can arise regarding ownership of data and consent for an individual’s data to be captured into a machine learning system, as well as security considerations when sharing data between institutions and AI systems. Because applied machine learning in paediatric neuroimaging and indeed health care as a whole is in its relative infancy, issues relating to data management, ownership and consent are likely to arise [39, 40].

## Artificial intelligence in paediatric neuro-oncology

Having reviewed the differing AI techniques that have been applied in paediatric brain imaging, we now examine the specific use case of paediatric neuro-oncology to inform the best suited roles for such methodologies in future clinical use.

### Diagnosis

Brain tumours represent the most common solid tumour reported in children [41]. Despite advances in diagnostic adjuncts, neurosurgical techniques, adjuvant treatment and supportive care, they remain the leading cause of paediatric cancer-related deaths [42]. Paediatric brain tumours present a significant challenge to treating clinicians for several reasons, including neurosurgical accessibility, lesional intimacy with surrounding critical structures, potential metastatic dissemination and the impact of therapy on the developing brain. Recently, these factors have been supplemented by an emerging awareness of biological heterogeneity within pre-defined tumour entities, leading to expanded tumour subgroupings and evolving risk stratification systems based upon this intra-tumoural molecular landscape [43–48]. These considerations are compounded by relatively small patient numbers when compared to either adult counterparts or non-central nervous system (CNS) tumours [41, 49, 50]. This presents a high-dimensional data problem: a deep, multi-faceted, feature-rich data set generated from a small number of patients to whom traditional statistical analysis is often not applicable, therein making accurate outcome prediction challenging [1].

The diagnosis of a childhood brain tumour is largely standardised historically; conventional MR imaging is acquired at presentation before proceeding to surgery in the form of either biopsy, debulk or resection, with tissue samples undergoing morphological assessment by a histopathologist. Over recent years, the advent of molecular techniques such as genomic arrays, gene expression profiling, fluorescent in situ hybridisation (FISH), and genome sequencing and methylomics has refined our understanding of paediatric brain tumours beyond simple histological classifications, identifying inherent subgroups within pre-defined tumour entities based upon shared genetic aberrations and biological behaviour [44–49, 51–54].

Noninvasive radiologic tumour assessment has also evolved in recent years through the advent and application of multi-parametric MRI, including diffusion-weighted imaging, perfusion-weighted imaging and MR spectroscopic sequences [55]. Modern advanced MRI is now capable of generating physiological and biochemical features of a lesion, producing detailed information beyond simple anatomical description [56]. Clearly, improved radiologic diagnostic accuracy at the time of clinical presentation is a vital adjunct to the treating clinician, facilitating improved planning regarding the necessity for aggressive surgery, the accuracy of information imparted to patients and parents, and the ability to plan adjuvant oncological therapy — all factors in which AI might provide additional data to aid in individual-case decision-making.

### Advanced multi-parametric magnetic resonance imaging techniques

Routine contrast-enhanced diagnostic MRI, whilst providing adequate anatomical description of a lesion, does not describe its physiological or metabolic behaviour. In addition, there is radiologic diagnostic overlap when assessing many childhood brain tumours because they can display similar T1, T2 and contrast-enhanced appearances. Neither is the extent of contrast enhancement in paediatric brain tumours indicative of tumour grade, unlike their adult counterparts [57]. Indeed, low-grade pilocytic astrocytomas (World Health Organization [WHO] grade I) enhance vividly, whilst *H3K27*-mutated diffuse midline gliomas of the pons (WHO grade IV) can display minimal contrast enhancement [58, 59].

It is hoped that multi-parametric MRI techniques will supplement conventional imaging data of brain tumours, providing more accurate, noninvasive in vivo radiophenotyping and radiologic determination of tumour molecular sub-groups where appropriate [55, 56, 60–70]. Indeed, diffusion-weighted imaging (DWI), perfusion-weighted imaging (PWI) and MR spectroscopy are now routinely acquired as part of the initial assessment of a child with a brain tumour.

### Radiogenomics in paediatric brain tumour imaging

It is anticipated that through such techniques, improved tumour characterisation might better inform prognosis when compared with standard imaging metrics such as tumour dimensional size or volume [1, 71–73]. Moreover, correlating advanced radiophenotypes with tumour molecular biology data, termed radiogenomics, might improve outcome prediction capabilities further. Radiogenomics studies have identified discrete imaging signatures for proven molecular tumour subgroups in brain tumours, including the most common paediatric malignant lesion, medulloblastoma — signatures that could have roles as predictive biomarkers [60–62, 66]. Taking this further, radiogenomics multi-institutional studies have demonstrated proof-of-concept results for the prediction of medulloblastoma subgroups from diagnostic MR imaging using AI [61, 62, 74].

### Artificial intelligence techniques in neuro-oncology imaging

#### Diagnosis

As discussed, there are instances where these machine learning techniques, now at the forefront of AI research in paediatric brain imaging, have been able to extract additional data from pre-existing imaging studies. Indeed, ANNs have had some success at predicting posterior fossa tumour types using such approaches to stratify pilocytic astrocytoma, medulloblastoma, ependymoma and diffuse midline glioma of the pons [75] using only T2-weighted imaging. Decision trees have also had some success in identifying prognostic factors for survival in recurrent adult glioblastoma multiforme (WHO grade IV glioma), as well as classifying between low- and high-grade gliomas [76, 77]. In children, the neuro-oncological success of these techniques has been more modest but still represents opportunity for future work. Considering LDAs, there has been some success in using ^1^H MR spectroscopy and DWI data from paediatric brain tumours so achieve successful categorisation of medulloblastomas, ependymomas, infiltrating gliomas and pilocytic astrocytomas [78–81].

Support vector machines have been used in the field of MRI texture analysis in brain tumours, an application of machine learning that quantifies imaging data to generate an image texture, a feature usually imperceptible to the human eye [82, 83]. Texture analysis provides value to the clinician because it makes use of the whole tumour imaging dataset and, as such, accounts for intra-tumour heterogeneity, which might not be captured by a single site or even multisite biopsy [83]. Texture analysis has successfully diagnosed and graded brain tumours, discriminating among pilocytic astrocytomas, medulloblastomas and ependymomas with an accuracy of up to 95% [83–86].

Building upon the use of texture analysis, Orphanidou-Vlachou et al. [87] combined the power of both LDA and a probabilistic neural network to classify posterior fossa tumours into medulloblastoma, pilocytic astrocytoma or ependymoma, with 86–93% accuracy based on their validation leave-one-out cross-validation of the techniques used. This application of AI in diagnostic assessment provides a future adjunct to diagnostic efficacy and interestingly can be performed on standard-acquisition T1- and T2-weighted imaging.

Support vector machines have also been successfully used to categorise tumour grade by combining conventional data with additional multi-parametric imaging information such as diffusion tensor imaging and PWI data [88]. In addition, they have been used to predict survival after surgery for adults with gliomas and metastases, with accuracy rates of up to 95%, utilising MRI and clinical, surgical resection and histopathological assessment data [89–93]. Li et al. [94] used a successful SVM approach to differentiate pilocytic astrocytomas from ependymomas on the basis of preoperative imaging, with a sensitivity of 93% and specificity of 80%, yielding an overall accuracy of 88%. Similar success has been seen through the use of naïve Bayesian classifiers, where they have successfully classified and graded brain tumours using multi-parametric MRI datasets [95, 96]. However, they have performed comparatively less well against other techniques such as SVMs and random forest classifiers.

#### Outcome prediction

**Fig. 3:**
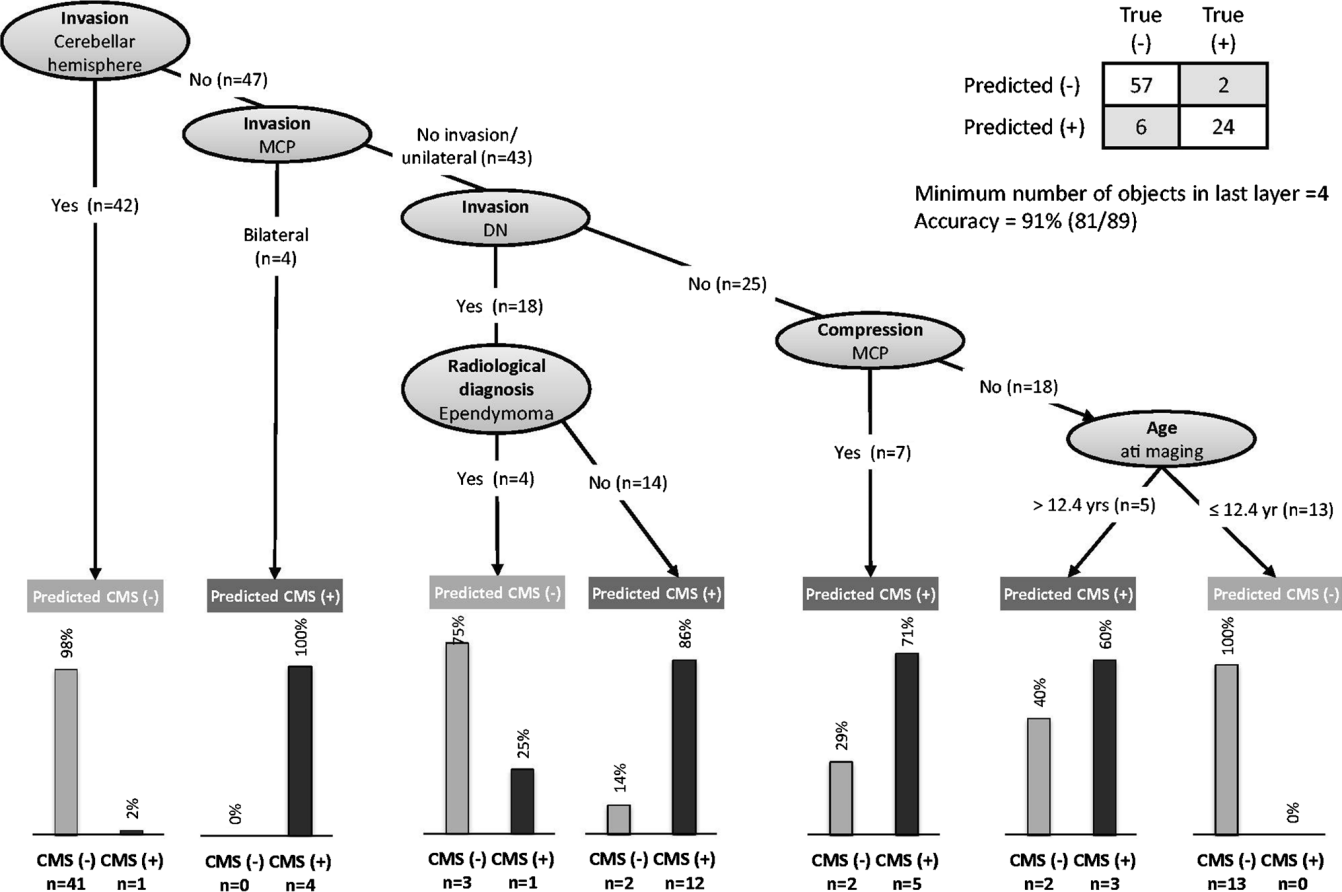
A C.45 decision tree with the highest accuracy (91.0%, 81/89) to predict cerebellar mutism syndrome (*CMS*). This decision tree was based on radiologic identification of specific imaging features including cerebellar hemisphere invasion, bilateral middle cerebellar peduncle (*MCP*) invasion, dentate nucleus (*DN*) invasion, preoperative radiologic diagnosis of ependymoma, MCP compression and age. *n* number, *yr* year. Reproduced from [97]

Whilst the majority of neuro-oncological uses of AI in brain imaging have focused on tumour stratification, exciting opportunities are presenting themselves in regard to outcome prediction. An exemplar of such an approach that harnesses the power of human/computer interaction is the work by Liu et al. [97] in predicting the risk of cerebellar mutism syndrome in children with posterior fossa tumours, based on their preoperative imaging and a C.45 (type of decision tree methodology) decision tree analysis (Fig. 3). This system used radiologist identification of the location of the tumour, invasion or compression of structures by the tumour, and age of the child to determine risk of cerebellar mutism syndrome, with a risk stratification accuracy of 88.8%. This type of approach has the power to significantly alter our discussions regarding prognostication with patients and their parents, as well as alerting the treating team to potential outcome issues preoperatively.

## Neuroimaging datasets

Rare diseases such as paediatric brain tumours often present diagnostic challenges, particularly if the presenting radiologic phenotype is incongruent from any established radiologic diagnostic pattern or the local radiologic team lacks specialist experience within a particular field. A means of improving radiologic diagnosis of rare disease is through contribution of imaging features to shared databases, allowing comparison of newly presenting imaging features against a larger, previously unavailable reference library.

Open-access public datasets are now widely available, including OpenNEURO.org (sharing platform for MRI and electrocardiogram data), the UK Biobank (large medical database including neuroimaging), the Enhancing NeuroImaging Genetics through Meta-analysis (ENIGMA) Consortium (collaborative network of large-scale studies from over 70 institutions) and the Human Connectome Project (neural data compilation). These resources aim to increase available sample sizes beyond that of a single institution, generating data sets that might allow investigation of previously inscrutable questions. The breadth of data available on OpenNeuro.org has contributed to a wide range of studies, including human brain connectome, deductive reasoning function in children, white matter disease in schizophrenia and bipolar disease, and automatic prediction of MRI image quality [98–101].When accessing such resources, care should be taken to check for updating datasets and processing pipelines and models, as well as ongoing quality control adjustments made by the host organisation; all of these factors can impact study outcomes [102–106].

Successful applications of specific paediatric radiologic databases include the INTERPRET (not an acronym) and Autism Brain Imaging Data Exchange (ABIDE) projects. The INTERPRET project successfully accrued ^1^H MR spectroscopy features of brain tumours, a feature that not all radiologists could interpret, forming the basis of a linear-discriminant-analysis-driven decision-support system. The INTERPRET decision support system aimed to be a universally accessible to physicians and biochemists, allowing the input of new case data from any MRI scanner. Accurate tumour classification was achieved in 69–85% of test set analyses, although the authors recommended further evaluation of classification performance limits of the model and encountered issues with data quality control in early model iterations [107, 108].

The ABIDE aggregated more than 1,000 functional MRI scans of 539 children with autistic spectrum disease and case-matched controls, identifying common features in regions of known dysfunction in autism spectrum disease (mid- and posterior insular and posterior cingulate cortex) and also regions less frequently evaluated. This shared database demonstrates the potential benefits of pooling multiple source datasets in confirming established features, and in the discovery of novel features of rare diseases [109].

Open-access shared data sets present data security and confidentiality issues, particularly in the context of rare diseases; re-anonymisation has been possible in datasets anonymised as per National Institutes of Health guidelines, leading to some collaborative efforts maintaining private-access databases [110–112].

## Summary

Multi-parametric MRI is becoming established within the imaging community for assessing paediatric brain tumours. The hope is that the feature-rich data generated will lead to improved radiomic feature extraction and, in certain instances, correlation with molecular subgroups. While radiogenomics and radiophenotyping studies are described, such data are not routinely integrated to enable up-front risk stratification and outcome prediction for children with brain tumours. Relapse and recurrence rates and time windows for such events have not been predicted using this approach. Artificial intelligence, underpinned by the machine learning techniques discussed here, offers a potential solution for enabling personalised medicine within this domain.

Artificial intelligence provides us with useful tools for handling large volumes of high-dimensional data generated from modern medical imaging practice. Applying machine learning models to paediatric brain multi-parametric MRI data and integrating the accompanying genetic, histopathological, clinical and surgical data should enable better risk-stratification systems, better prognostic prediction and individualised surveillance protocols for children depending on their underlying diagnosis. The clinical translation of such systems is occurring through the implementation of the machine learning techniques described here and the AI underpinning clinical decision-support systems. A clinical decision-support system is defined as any piece of software that takes, as input data, the information about a clinical situation and produces, as output, the inferences regarding the clinical situation that can assist practitioners with their decision-making [113]. Clinical decision-support systems, and the machine learning techniques underpinning them, are gradually becoming accepted into medical application in view of both their data handling capabilities and predictive outputs [113, 114], particularly because the volume of information assimilated in modern medical practice (as is evidenced by the large volumes of imaging data generated in a modern paediatric neuro-imaging study) can be potentially prohibitive to human decision-making performance [115, 116].

We predict that validation and integration of neuroimaging AI applications into mainstream clinical practice will occur in two stages. First, classification and outcome prediction clinical decision support systems will be trialed in parallel to established clinical practices such as neuro-oncology multi-disciplinary team meetings, assessing performance in direct comparison to local expert clinical opinion. Second, models that demonstrate potential will then be prospectively trialed with unseen data under the conditions of a formal trial. With respect to paediatric neuroimaging applications, validation of applications might be a long-term process in view of small patient cohorts and sporadic presentations of certain conditions. There is also the challenge of integrating such systems into existing health informatics infrastructure; at present most National Health Service (NHS) systems are not capable of incorporating such models, nor are they conducive to automated data collection. The Topol Report has advised focused funding for digital transformation within the NHS to aid in development of such projects.

Meaningful contributions to the field can be made at a local level through the contribution of well-organised data sets of rare disease imaging, particularly where advanced metric imaging has been performed. Data should ideally be contributed to secure open-access databases that encourage collaborative work in rare-disease fields. Collaborative initiatives should be encouraged between clinicians and data scientists to ensure application of appropriate data handling and machine learning algorithms, and the development of clinician-led AI projects within health care.

In the next decade, we expect to see the gradual acceptance and deployment of such clinical diagnosis decision support systems into clinical paediatric neuroradiology as they are translated from the academic domains discussed here to the workstation and, most important, become valuable tools for use in routine clinical practice.
